# Socioeconomic inequalities in diabetes prevalence: the case of South Africa between 2003 and 2016

**DOI:** 10.1186/s12889-023-15186-w

**Published:** 2023-02-14

**Authors:** Sahar Sidahmed, Siegfried Geyer, Johannes Beller

**Affiliations:** Medical Sociology Unit, Hannover Medical School, Carl-Neuberg-Str. 1, 30625 Hannover, Germany

**Keywords:** Diabetes, Prevalence, South Africa, Social inequalities, DHS, Educational level

## Abstract

**Background:**

Diabetes is a growing epidemic worldwide and the effect of socioeconomic status (SES) is frequently acknowledged in the literature. This study aims to compare the effect of SES on diabetes prevalence in South Africa between 2003 and 2016. In addition, vulnerable groups regarding diabetes development in 2016 will be identified.

**Methods:**

Using DHS data there were 8,006 participants (59.19% women) in 2003 and 10,292 participants (59.42% women) in 2016. Logistic regression and odds ratios (ORs) with 95% confidence intervals (CIs) were calculated for diabetes by age, gender, educational level and place of residence. To identify vulnerable groups with high risk of developing diabetes in 2016, the method of p-value based regression tree analysis was applied using “wealth index” and “weight perception” as additional variables.

**Results:**

There was an increase in diabetes prevalence from 3.86% in 2003 to 4.46% in 2016. Women had more risk of developing diabetes at both time points (27% in 2003 and 24% in 2016 more risk). Increase in age and living in urban areas were associated with more risk of developing diabetes at both time points. There was no specific pattern regarding risk of developing diabetes and educational level in case of women. However, men who completed secondary school or had a higher diploma or above had more risk of developing diabetes in 2016 (OR = 2.24 and 4.67 respectively). Vulnerable groups who have higher risk of developing diabetes in 2016 were participants aged “60 years or older” with a wealth index of “rich” or “richer”, followed by participants from the same age group who were “poor” or “poorer” and participants aged “40–59 years” with a wealth index of “rich” or “richer”. Subsequently were participants from the age group “15–39 years” with a weight perception of “overweight” or “obese”.

**Conclusion:**

Diabetes prevalence increased in South Africa between 2003 and 2016. Main risk factors were age, gender and living in urban areas. Men with high educational level were more at risk of developing diabetes in 2016. Vulnerable groups in 2016 were participants 40 years and older, particularly with high SES. This was followed by younger participants who were obese or overweight.

## Background

Diabetes is one of the world’s fast-growing epidemics and the ninth leading cause of death around the world [1]. According to the International Diabetes Federation (IDF), 10.5% of the world’s adult population (aged 20–79 years) had diabetes in 2021 and this is expected to reach 12.2% in 2045. Moreover, diabetes also contributed to 6.7 million deaths among adult people around the world in 2021 [2]. Population aging and changes in lifestyle due to increased urbanization are the main attributing factors to the rise in diabetes prevalence. Population aging is responsible for about 16% of predicted increase in diabetes prevalence world-wide [2]. Living in urban areas is usually associated with a more sedentary lifestyle and higher consumption of unhealthy food and hence high diabetes prevalence [3].

Although the rise in diabetes prevalence is occurring around the world, Africa has yet the lowest prevalence compared to other regions [2]. However, due to growing shift towards more urbanization associated with unhealthy diet and low physical activity, the number of people with diabetes is expected to increase in this Region from 24 million in 2021 to 55 million people in 2045 with a 129% increase [2]. South Africa is considered one of the major transitioning economies and ranked one of the upper-middle-income countries according to the world bank [4]. Furthermore, it is one of the countries experiencing the epidemiological shift from communicable to non-communicable diseases (NCDs) [5]. According to the WHO, 51% of deaths in South Africa are due to NCDs [6]. It also suffers from the double burden of infectious and noninfectious conditions like tuberculosis, diabetes, cerebrovascular diseases, HIV, hypertension, influenza, and pneumonia. While there is a decline in tuberculosis, the leading cause of death in South Africa, there is an increase in diabetes prevalence, the second leading cause of death [5].

South Africa also had the highest number of people with diabetes (aged 20–79 years) in the African Region with an increase from 1.9 million in 2011 to 4.2 million people in 2021 [2]. Risk factors related to developing diabetes such as economic development and increased urbanization associated with unhealthy diet and physical inactivity are present in South Africa [7]. Dietary consumption of foods with excessive sugar, fat and salt contributed to remarkably high level of obesity in South Africa. Apart from the fact that, 38.8% of men and 69.3% of women in South Africa are overweight or obese [8], South African women had the highest documented prevalence of obesity in sub-Saharan Africa [9]. According to the Demographic and Health Surveys (DHS) report in 2003, 76% of men and 86% of women participating in the survey in South Africa were physically inactive [10]. Furthermore, it is estimated, that 38% of people with diabetes in South Africa are undiagnosed [11].

In addition to previously mentioned risk factors, the effect of socioeconomic status (SES) on developing diabetes is very evident. In 1981, Mueller and Parcel defined SES as “the relative position of a family or individual on a hierarchical social structure, based on their access to or control over wealth, prestige and power” [12]. Health inequalities have been explained in relation to unequal burden of disease or behavioral risk factors that affect certain subgroups of the population [13]. Recently, Healthy People 2010 defined health disparities as: “differences that occur by gender, race or ethnicity, education or income, disability, geographic location, or sexual orientation” (p. 11) [14]. An association between diabetes prevalence and socioeconomic inequalities has often been presented in the literature with varying results reported from different countries around the world [11, 15–18]. Studies from high-income countries usually associate diabetes prevalence with low socioeconomic groups [15–18]. However, research done in low- and middle-income countries (LMIC) implies a higher prevalence among people with high socioeconomic status [11, 19, 20].

Generally, there are three indicators used in studying health inequalities related to SES which are: education, occupation and income [21]. These variables are usually used together or separately. Choosing which variable to evaluate SES inequality depends on the study population and outcome, in addition to data availability [22–24].

## Objectives

Although South Africa has the highest number of people with diabetes in the African Region [2], there is scarcity in literature investigating socioeconomic inequalities in relation to diabetes prevalence. Ataguba, Akazili and McIntyre., studied socio-economic related health inequality in South Africa between 2002 and 2008, using a set of common assets and household characteristics to create composite indices of socio-economic status. They found that diabetes is more common among the rich, yet it is increasingly reported among poor people [25]. Mutyambizi et al., investigated lifestyle factors influence on diabetes inequalities in South Africa in 2012, and stated that self-reported diabetes is higher among rich people [11].

Using the Demographic and Health Surveys (DHS) Program database, this study aims to compare between diabetes prevalence and the existence of related socioeconomic inequalities at two time points (2003 and 2016). Furthermore, it will explore risk factors related to diabetes development at each time point and identify vulnerable groups in 2016. Vulnerable groups are people who have higher risk of developing diabetes in South Africa in 2016. The present study will provide evidence-based literature for upcoming research by identifying risk factors and the characteristics of people or groups with diabetes in South Africa. This will help in health care planning, preparing group-oriented prevention programs and raising awareness in the society, and hence, eliminating social inequalities.

**Research questions**.

1. Is there a difference in the prevalence of diabetes in South Africa between 2003 and 2016?
2. Are there any social inequalities regarding diabetes prevalence in 2003 and 2016?
3. Is there a difference between 2003 and 2016 in the association of SES with risk of developing diabetes?
4. What are the vulnerable groups who are at risk of developing diabetes in 2016?

## Methods

### Data

This study was done using the Demographic and Health Surveys (DHS) data. DHS program started in 1984 by giving technical assistance to more than 400 surveys in over 90 countries and helped improve global understanding of health and population trends in developing countries. It collects and distributes nationally representative data on fertility, family planning, maternal and child health, gender, HIV/AIDS, malaria, and nutrition [26].

### South Africa DHS (SADHS)

#### SADHS 2003

The SADHS 2003 sample was designed to be a nationally representative probability sample; hence the country was stratified into nine provinces and each province was further stratified into urban and non-urban areas. Statistics South Africa (Stats SA) provided the sampling frame for the SADHS based on the enumeration areas (EAs) list of approximately 86 000 EAs created during the 2001 census. Then, the systematic sampling of households/stands from the selected EAs was done. In 2003, 7,041 women aged 15–49 years were interviewed using The Women’s Questionnaire and 3,118 men aged 15–49 years were interviewed using The Men’s Questionnaire. In addition, 8 115 adults aged 15 years and above were interviewed using the Adult Health Questionnaire [10].

The main objective of the SADHS 2003 was to deliver data on: households and respondents characteristics, contraception and fertility, sexual behavior, HIV and AIDS, infant and child mortality, maternal and child health, infant and child feeding, adolescent health, mortality and morbidity in adults, utilization of health services, adult health, risk factors for chronic diseases, oral health and health of older persons [10].

#### SADHS 2016

The Statistics South Africa Master Sample Frame (MSF) was used for the SADHS 2016 which was created using Census 2011 enumeration areas (EAs). In this frame, EAs of manageable size were treated as primary sampling units (PSUs), while small neighboring EAs were joint together to form new PSUs, and large EAs were split into conceptual PSUs. There was also information about the geographic type (urban, traditional, or farm) and the probable number of residential dwelling units (DUs) in each PSU. A stratified two-stage sample design was used in the SADHS 2016. The first stage included a probability proportional to size sampling of PSUs and the second stage involved systematic sampling of DUs. In 2016, 8,514 women, 3,618 men and 10,336 adults were interviewed [27].

In addition to previously mentioned objectives of the 2003 SADHS, the 2016 SADHS collected information on breastfeeding practices, nutrition and physical and sexual violence against women. A key objective of the SADHS 2016 was to collect data about use of tobacco, alcohol, and codeine-containing medications. It also provided information on the prevalence of anemia among children aged 6–59 months and the prevalence of hypertension, anemia, high HbA1c levels (an indicator of diabetes), and HIV among adults aged 15 years and older [27].

### Definition of diabetes cases

SADHS was an interview survey; hence case definition was based on self-reported information. To identify people with diabetes, participants were asked, “Has a doctor or nurse or health worker told you that you have or have had diabetes or blood sugar?” If the response was “Yes,” the person was classified as having diabetes. Persons responding “No” were classified not to have diabetes. Participants who responded, “don’t know” (0.78% in 2003 and 0.43% in 2016) and “missing” cases (0.09% in 2003) were excluded from the statistical analysis. There were no missing cases in 2016. The question did not imply whether it is type 1 or type 2 diabetes.

### Independent variables

Participants were in the age between 15 and 95 years at the time of interview. They were classified into three age groups: 15–39 years, 40–59 years and 60 years and older. Based on the highest achieved level of education, there were four categories: no education (no school diploma), complete primary education (completed 6 years of primary school), complete secondary education (completed 6 years of secondary school) and higher education (higher diploma or more). Place of residence was classified into rural and urban areas. For identifying vulnerable groups in 2016, two additional variables were used, “wealth index” and “weight perception”. Wealth index is a score given according to household’s belongings such as television, bicycle or car, materials used for housing and source and access to water and toilet facilities. Using principal components analysis, households are located on a continuous scale of relative wealth. All interviewed households are divided into five wealth quintiles [27, 28]. Wealth index has five categories poorest, poorer, middle, richer and richest. For the “weight perception” variable, participants were asked “Do you personally think you are underweight, normal weight, overweight, or obese?” and accordingly there were five categories, underweight, normal weight, overweight, obese and do not know. Vulnerable groups were not explored in 2003 due to lack of additional variables: “wealth index” and “weight perception” in the 2003 dataset.

### Statistical analysis

To answer the research questions, analyses were performed in five steps. The first step included descriptive analyses for all independent variables and calculating diabetes prevalence. In the second step, diabetes prevalence was predicted using logistic regression and using age, gender, educational level and place of residence as predictors. Results were reported as odds ratios (ORs) with 95% confidence intervals (CIs). This was repeated separately for men and women. The first two steps were done for the 2003 and 2016 datasets individually.

The third step was analyzing time period differences in diabetes prevalence; hence the two datasets were combined using the SADHS 2003 as reference. Logistic regression and odds ratios (ORs) with 95% confidence intervals (CIs) were then calculated for diabetes by age, gender, education and place of residence. In the fourth step, effect of SES on the prevalence of diabetes was assessed using education by repeating logistic regression analysis for each category of educational level for men and women separately. These four steps were done using STATA.

Finally, to identify vulnerable groups with high risk of developing diabetes in 2016, the method of p-value based regression tree analysis was applied using “wealth index” and “weight perception” as additional variables. Classification and regression tree (CART) is a prediction method that is usually used with dichotomous outcomes preventing the assumptions of linearity. In this technique, the sample is repeatedly divided into subgroups according to the value of one of the predictor variables. The result is a group of branches creating a treelike structure with each final branch providing a yes/no prediction of the outcome [29, 30].

## Results

### Characteristics of study population

Study population consisted of 8,006 participants (59.19% women) in 2003 and 10,292 participants (59.42% women) in 2016. More than half the participants (59.86% in 2003 and 56.64% in 2016) were in the age group 15–39 years. Two thirds of the participants completed secondary school (64.98% in 2003 and 64.56% in 2016). There was a decrease in the percentage of participants without education from 12.45% in 2003 to 8.42% in 2016. Other than a small difference in the percentage of men and women without education -women had a higher percentage-, no significant gender differences were found in level of education. 57.32% of participants in 2003 lived in urban areas compared to 55.04% in 2016. No gender differences were found regarding place of residence. Frequencies for population characteristics are presented in (Table 1).

**Table 1:** Population characteristics stratified by gender and time point

	2003		2016	
	Men N (%)	Women N (%)	Men N (%)	Women N (%)
**N**	3,267 (40.81)	4,739 (59.19)	4,176 (40.58)	6,116 (59.42)
**Age group**				
15–39 years	2,067 (63.27)	2,725 (57.50)	2,560 (61.30)	3,269 (53.45)
40–59 years	827 (25.31)	1,356 (28.61)	1,031 (24.69)	1,732 (28.32)
60 + years	373 (11.42)	658 (13.88)	585 (14.01)	1,115 (18.23)
**Educational level**				
No education	331 (10.13)	666 (14.05)	283 (6.78)	584 (9.55)
Complete primary	502 (15.37)	719 (15.17)	781 (18.70)	1,046 (17.10)
Complete secondary	2,179 (66.70)	3,023 (63.79)	2,723 (65.21)	3,922 (64.13)
Higher	255 (7.81)	331 (6.98)	389 (9.32)	564 (9.22)
**Residence**				
Rural	1,348 (41.26)	2,069 (43.66)	1,866 (44.68)	2,761 (45.14)
Urban	1,919 (58.74)	2,670 (56.34)	2,310 (55.32)	3,355 (54.86)

### Diabetes prevalence

There was an increase in diabetes prevalence from 3.86% in 2003 to 4.46% in 2016. Diabetes was more prevalent in women at both time points. In 2003, 4.30% of women had diabetes compared to 3.21% of men. Percentage of women with diabetes was 5.05% in 2016 compared to 3.59% of men. The highest percentage of diabetes was found in the age group 40–59 years (48.57% of men and 45.10% of women) in 2003. However, it was highest among participants from the age group 60 years and older (58.00% of men and 48.54% of women) in 2016.

### Social inequalities in diabetes prevalence

The highest percentage of participants with diabetes at both time points was seen in men (60.0% in 2003 and 54.0% in 2016) and women (48.53% in 2003 and 44.66% in 2016) who completed secondary school. Most participants having diabetes lived in urban areas at both time points with no observable gender differences. This was more prominent in 2003 (75.24% of men and 71.08% of women) compared to 2016 (66.67% of men and 59.22% of women). (Table 2)

After applying logistic regression, significant effects were found in age and place of residence at both time points. There was a consistent positive relationship between age and diabetes prevalence at both time points for both men and women. However, it was more prominent in 2016 where the risk of developing diabetes for participants aged 60 years and older was relatively high (OR = 41 and 20 for men and women respectively). Risk of developing diabetes was more in women compared to men at both time points (27% in 2003 and 24% in 2016 more risk). Living in urban areas was also associated with more risk of developing diabetes at both time points. Nevertheless, it was more distinct in 2003 (OR = 2.14 and 2.12 for men and women respectively). No gender differences were observed regarding place of residence. Higher education was associated with lower risk of developing diabetes in 2003 but was a risk factor for developing diabetes in 2016. Results were statistically significant for participants who completed their secondary education or who had a higher diploma or above in 2016. There was no specific pattern regarding risk of developing diabetes and educational level in case of women. On the other hand, men who completed secondary school or had a higher diploma or above have shown a significant risk (p-value < 0.001) of developing diabetes in 2016 (OR = 2.24 and 4.67 respectively). (Table 3)

**Table 2:** Diabetes prevalence in 2003 and 2016 stratified by age, education and place of residence

	2003				2016			
	Diabetes				Diabetes			
	No		Yes		No		Yes	
	**Men N (%)**	**Women N (%)**	**Men N (%)**	**Women N (%)**	**Men N (%)**	**Women N (%)**	**Men N (%)**	**Women N (%)**
**N**	3,162 (96.79)	4,535 (95.70)	105 (3.21)	204 (4.30)	4,026 (96.41)	5,807 (94.95)	150 (3.59)	309 (5.05)
**Age group**								
15–39 years	2,042(64.58)	2,689 (59.29)	25 (23.81)	36 (17.65)	2,545 (63.21)	3,243 (55.85)	15 (10.00)	26 (8.41)
40–59 years	776 (24.54)	1,264 (27.87)	51 (48.57)	92 (45.10)	983 (24.42)	1,599 (27.54)	48 (32.00)	133 (43.04)
60 + years	344 (10.88)	582 (12.83)	29 (27.62)	76 (37.25)	498 (12.37)	965 (16.62)	87 (58.00)	150 (48.54)
**Educational level**								
No education	317 (10.03)	626 (13.80)	14 (13.33)	40 (19.61)	271 (6.73)	530 (9.13)	12 (8.00)	54 (17.48)
Primary	483 (15.28)	666 (14.69)	19 (18.10)	53 (25.98)	751 (18.65)	961 (16.55)	30 (20.00)	85 (27.51)
Secondary	2,116 (66.92)	2,924 (64.48)	63 (60.00)	99 (48.53)	2,642 (65.62)	3,784 (65.16)	81 (54.00)	138 (44.66)
Higher	246 (7.78)	319 (7.03)	9 (8.57)	12 (5.88)	362 (8.99)	532 (9.16)	27 (18.00)	32 (10.36)
**Residence**								
Rural	1,322 (41.81)	2,010 (44.32)	26 (24.76)	59 (28.92)	1,816 (45.11)	2,635 (45.38)	50 (33.33)	126 (40.78)
Urban	1,840(58.19)	2,525 (55.68)	79 (75.24)	145 (71.08)	2,210 (54.89)	3,172 (54.62)	100 (66.67)	183 (59.22)

**Table 3:** Odds ratios of diabetes prevalence in 2003 and 2016 stratified by age, education and place of residence for men and women as estimated by means of logistic regression

	Diabetes											
	2003						2016					
	Men n = 3267			Women n = 4739			Men n = 4176			Women n = 6116		
	**OR**	**P**	**95% CI**	**OR**	**P**	**95% CI**	**OR**	**P**	**95% CI**	**OR**	**P**	**95% CI**
**Age group** 15–39 years (Ref.)												
40–59 years	5.55	< 0.001	3.38–9.10	5.40	< 0.001	3.60–8.10	9.18	< 0.001	5.10-16.55	10.45	< 0.001	6.80-16.06
60 + years	8.61	< 0.001	4.73–15.70	10.93	< 0.001	6.99–17.08	41.09	< 0.001	23.09–73.13	20.93	< 0.001	13.38–32.72
**Educational level** No education (Ref.)												
Primary	1.20	0.628	0.580 − 2.46	1.55	0.050	1.00-2.41	1.75	0.116	0.87-3.51	1.20	0.323	0.83-1.73
Secondary	1.47	0.247	0.764 − 2.85	1.25	0.302	0.89-1.91	2.24	< 0.001	1.62–6.05	1.18	0.378	0.82-1.69
Higher	1.31	0.562	0.529 − 3.23	1.30	0.460	0.65-2.63	4.67	< 0.001	2.22–9.85	1.50	0.102	0.92-2.45
**Residence** Rural (Ref.)												
Urban	2.14	0.001	1.34–3.42	2.12	< 0.001	1.53–2.94	1.39	0.089	0.95-2.02	1.29	0.043	1.01–1.66

### Difference in the effect of SES on diabetes prevalence between 2003 and 2016

After merging the two datasets using 2003 dataset as reference, there was almost no difference in risk of developing diabetes between 2003 and 2016 (OR = 1.02). (Table 4) The same was noticed after repeating logistic regression for men and women separately. Age effect was observed for both men and women. However, men at the age of 60 years and older had a higher risk (OR = 21.67) of having diabetes in 2016 compared to women (OR = 15.32) from the same age group. While the effect of educational level had no specific pattern regarding risk of developing diabetes in women, it was a clear risk factor for men in 2016. Men who completed secondary school (OR = 2.37) or had a higher diploma or above (OR = 3.04) had a greater risk of having diabetes in 2016 in comparison to 2003. Participants living in urban areas had a higher risk of developing diabetes in 2016. This applies for both men (OR = 1.66) and women (OR = 1.56). (Table 5)

**Table 4:** Odds ratios of time effect on diabetes prevalence stratified by age, education and place of residence as estimated by means of logistic regression

	Diabetes		
	OR	P	95% CI
**2.wave**	1.02	0.810	0.87–1.19
**Age group**			
15–39 years (Ref.) ^a^	-	-	-
40–59 years	7.46	< 0.001	5.93–9.38
60 + years	17.39	< 0.001	13.65–22.15
**Gender**			
Men (Ref.) ^a^	-	-	-
Women	1.25	0.005	1.07–1.47
**Educational level**			
No education (Ref.) ^a^	-	-	-
Complete primary	1.38	0.009	1.08–1.77
Complete secondary	1.50	0.001	1.18–1.90
Higher	1.89	< 0.001	1.38–2.59
**Residence**			
Rural (Ref.) ^a^	-	-	-
Urban	1.59	< 0.001	1.36–1.88

^*a*^ This is the reference group used in the logistic regression. Hence, no Odds ratio could be computed to this group, since the other groups are compared to it.

**Table 5:** Odds ratios of time effect on diabetes prevalence stratified by age, education and place of residence for men and women as estimated by means of logistic regression

	Diabetes					
	Men n = 7443			Women n = 10,855		
	**OR**	**P**	**95% CI**	**OR**	**P**	**95% CI**
**2.wave**	0.99	0.934	0.76–1.29	1.04	0.704	0.86–1.25
**Age group**						
15–39 years (Ref.) ^a^	-	-	-			
40–59 years	7.01	< 0.001	4.82–10.20	7.57	< 0.001	5.66–10.12
60 + years	21.67	< 0.001	14.67–31.99	15.32	< 0.001	11.25–20.87
**Educational level**						
No education (Ref.) ^a^	-	-	-	-	-	-
Complete primary	1.52	0.098	0.92 − 2.50	1.36	0.032	1.03–1.80
Complete secondary	2.37	< 0.001	1.49–3.77	1.23	0.135	0.94–1.63
Higher	3.04	< 0.001	1.75–5.30	1.48	0.051	1.00–2.21
**Residence**						
Rural (Ref.) ^a^	-	-	-	-	-	-
Urban	1.66	0.001	1.24–2.21	1.56	< 0.001	1.28–1.90

^*a*^ This is the reference group used in the logistic regression. Hence, no Odds ratio could be computed to this group, since the other groups are compared to it.

### Vulnerable groups at risk of developing diabetes in 2016

**Fig. 1:**
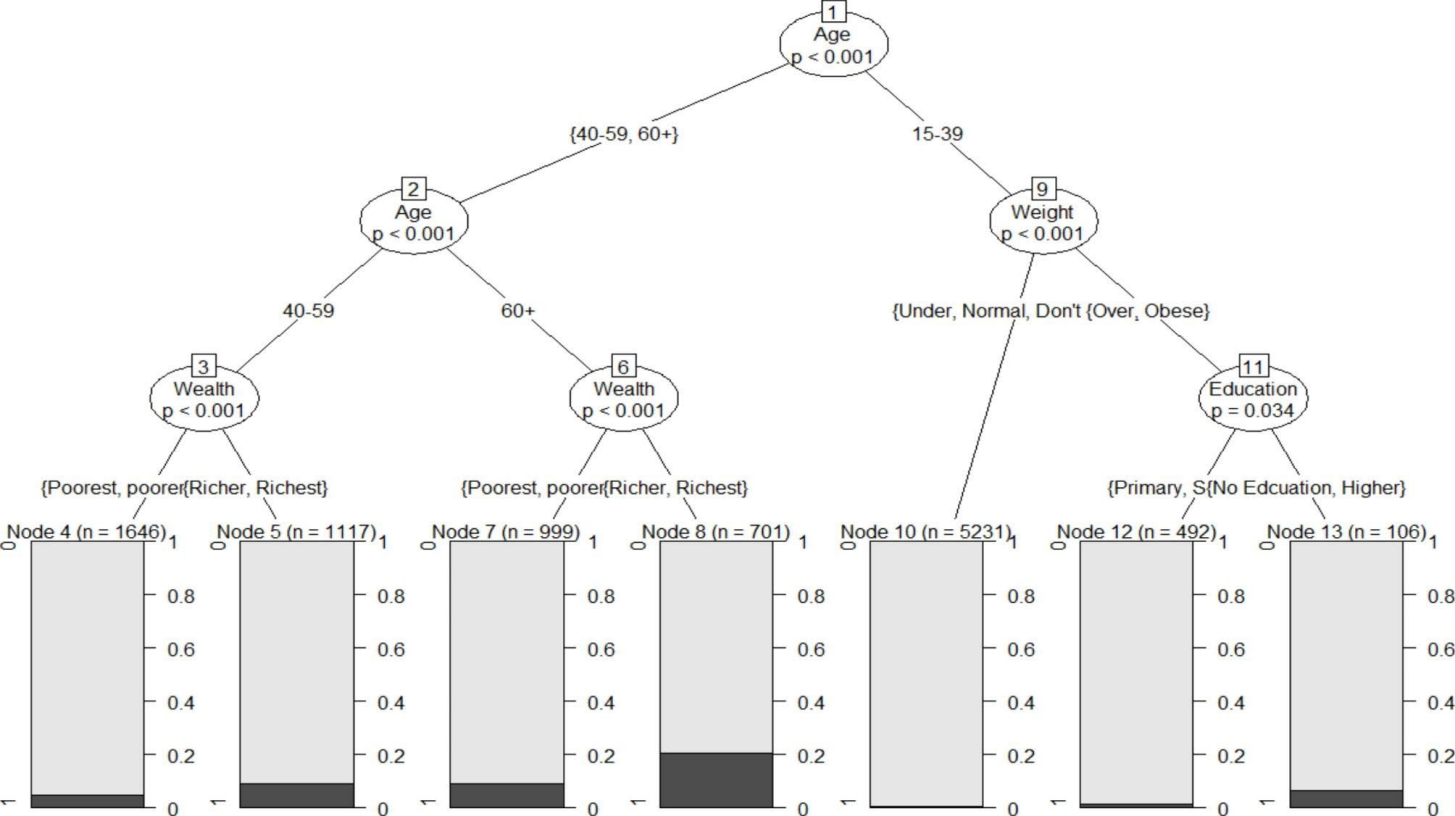
Results from the regression tree analysis predicting vulnerable groups at risk of developing diabetes in 2016

The method of p-value based regression tree analysis was used to identify vulnerable groups who have higher risk of developing diabetes in 2016. Hence, participants who were “60 years and older” with a wealth index of “rich” or “richer” were the most vulnerable group identified, followed by participants from the same age group with a wealth index of “poor” or “poorer” and participants who were “40–59 years” with a wealth index of “rich” or “richer”. Subsequently were participants from the age group “15–39 years” with a weight perception of “overweight” or “obese” and with educational level of “no education” or “high education” followed by participants who were “40–59 years” with a wealth index of “poor” or “poorer”. Subgroups with the lowest risk of developing diabetes were participants from the age group “15–39 years” with a weight perception of “underweight”, “normal weight” or “don’t know” and participants from the same age group with a weight perception of “overweight” or “obese” and with educational level of “primary” or “secondary”. (Fig. 1).

## Discussion

### Diabetes prevalence and its association with SES in 2003 and 2016

This study investigated difference in diabetes prevalence and associated socioeconomic inequalities in South Africa between 2003 and 2016. In accordance with the literature, diabetes prevalence was higher in 2016 compared to 2003, with age, gender (women were more at risk), and living in urban areas being the main risk factors at both time points. Yet, the extent of the association of these risk factors with diabetes prevalence was different between 2003 and 2016. On the other hand, the effect of educational level was not consistent at both time points. There was however an indication of increased risk of diabetes with higher educational level in 2016, which was more prominent in men.

According to the IDF report, diabetes prevalence has indeed increased in South Africa from 3.4% in 2003 to 5.4% in 2017 [31, 32]. Although diabetes prevalence is increasing in Africa, the pattern of this increase is diverse among African countries. While the increase was minimal in Botswana from 3.6% in 2003 to 3.8% in 2017, it was very distinct in Gabon from 2.9% in 2003 to 7.0% in 2017 [31, 32]. In Brazil diabetes prevalence increased from 5.5% in 2006 to 8.9% in 2016 [33]. Though South Africa was the second African country after Ethiopia with the highest number of people with diabetes in 2017, it became the first in 2021 [2, 32]. Increased urbanization associated with physical inactivity and unhealthy diet leading to obesity, in addition to tobacco use and harmful consumption of alcohol are considered risk factors contributing to the increase in diabetes prevalence [34].

Global reports indicate the association between age and diabetes development [2, 31, 32]. Whereas participants aged 40–59 years followed by participants aged 60 years and older had the highest diabetes prevalence for both men and women in 2003, diabetes prevalence increased with age for both genders in 2016. Lower prevalence of diabetes in participants aged 60 years and older in 2003 could be due to high levels of undiagnosed diabetes. As previously mentioned, 38% of diabetics in South Africa are undiagnosed [11]. In a study done in Botswana on patients admitted with acute heart failure, they found that almost half of them had undiagnosed diabetes [35]. Moreover, the highest percentage of undiagnosed diabetes is in Africa (53.6% of adults aged (20–79 years)) [2].

Gender variations in diabetes prevalence were frequently discussed in the literature. Although few studies reported that diabetes prevalence was higher in men [36, 37], most studies found women more at risk of developing diabetes [31, 38, 39]. Consistent with majority of the studies, results of this study revealed a higher diabetes prevalence among women at both time points. Possible reason for this could be the high level of obesity among South African women. It was stated in the literature that 69.3% of women in South Africa are overweight or obese compared to 38.8% of men [8, 9]. Research done in Nigeria [40], Uganda[38] and some west African countries, indicates that women reportedly had higher levels of obesity compared to men [41]. Obesity is directly linked to developing diabetes, especially in women [24, 42, 43]. In a study done by Mutyambizi et al., they found that obesity contributed to 24.8% of self-reported diabetes in South Africa [11].

Place of residence is one of the factors that contribute to inequalities in diabetes prevalence [2, 31, 32, 39]. Living in urban areas was frequently related to higher risk of developing diabetes, yet there are reported gender differences in studies done in some countries like Ghana[44] and Cameron [45]. In the present study, developing diabetes at both time points was more prominent in participants living in urban areas in comparison to participants living in rural areas. This was more evident in 2003 compared to 2016, and in men more than women. It is usually acknowledged that diabetes prevalence is higher in urban areas due to sedentary lifestyle and decreased physical activity. Though this is true in many cases, some authors identify other reasons. In studies done in India[46] and Bangladesh [47], they indicated that living in urban areas was associated with more awareness about diabetes and hence higher prevalence [46]. Likewise, in Cameron, Mbanya et al., found that undiagnosed diabetes was more in rural areas compared to urban areas [45]. Other explanations for this could be that rural areas are usually associated with inadequate resources, difficulty in access to healthcare services and prioritization of other health issues [2].

Socioeconomic inequalities and its association with diabetes prevalence were discussed in the literature with diverse results. While most of the studies done in USA[48], Canada[22] and western Europe [24] indicate that higher SES (high educational level, occupation and income) was associated with lower level of diabetes prevalence, studies done in low- and middle-income countries did not always show similar results.[18, 20] Results of this study show that diabetes prevalence had witnessed a steady increase with increased educational level and reached the highest level in participants who had secondary education. However, it exhibited a noticeable decrease in participants with higher educational level especially in 2003 and in women. Gender differences regarding SES effect on diabetes prevalence were also registered in the literature. Women were usually more affected by their SES and the higher it was the lower was their risk of developing diabetes compared to men. [49–51] Addo et al., did a study on Ghanian adults residing in Europe based on data derived from the multicenter Research on Obesity and Diabetes among African Migrants (RODAM). They found that diabetes prevalence decreased with increasing educational level in Ghanaian men and women in Europe and in men in urban Ghana; however, it increased with increasing level of education in men and women in rural Ghana. [44]

### Difference between 2003 and 2016 in the effect of SES on risk of developing diabetes

When examining the overall risk of developing diabetes in 2016 compared to 2003, there was no substantial difference found. The aforementioned risk factors of age, gender and urbanization were still present; however, high educational level was a distinct risk factor in 2016. Furthermore, assessing the effect of these risk factors on men and women separately revealed evident gender differences. Men aged “60 years and older” and the ones with high educational level were noticeably more at risk of developing diabetes in 2016 compared to women from the same categories. Similar results were found in a study done in west African countries, where older men were more at risk of developing diabetes than women.[52] In addition, Seiglie et al., studied diabetes prevalence in 29 low- and middle-income countries, and found that diabetes risk increased with increased educational level.[20] An explanation for this could be low level of physical activity associated with easy access to unhealthy diets and general sedentary lifestyle. Wu et al., did a study on adults aged 50 years and older from China, Ghana, India, Mexico, the Russian Federation and South Africa. The highest prevalence of low physical activity and obesity was in South Africa. [53] Furthermore, decline in physical activity with age is usually more prominent in men than women.[54, 55] It is not uncommon for studies done in high-income countries, such as, Canada,[56] Germany,[24] and Australia,[42] to link diabetes prevalence with lower SES. However, studies from low- and middle-income countries usually present contrasting results. Link between high income and high educational level with greater diabetes risk was stated in the literature.[20] Although the relation between diabetes risk and educational level was not merely linked to higher BMI,[57] obesity and physical inactivity are still major contributors.

### Vulnerable groups who are at risk of developing diabetes in 2016

Results from the present study, affirmed that, participants aged 40 years and older have higher risk of developing diabetes and this risk increases at age 60 years and older. Participants aged 40 years and older have an additional risk factor, namely, “wealth status”. Subsequently, being rich or richer increases the risk factor of developing diabetes. Mutyambizi et al., indicate that self-reported diabetes in South Africa is more common among rich people while undiagnosed diabetes is usually found among the poor.[11] Whereas some studies associate between higher diabetes prevalence and lower SES especially in women, [22, 48, 49] others link between higher diabetes prevalence and rich men with high SES.[58, 59] Additionally, men with lower SES in South Africa usually work in jobs that necessitate physical activity.[58] Being rich is associated with high diabetes prevalence not only because it is usually associated with sedentary lifestyle and unhealthy diet, but also because it is sometimes associated with higher level of awareness about diabetes[47] and easier access to healthcare facilities.[2].

On the other hand, younger participants aged 15–39 years who have a “weight perception” of overweight or obese have higher risk of developing diabetes compared to participants from the same age group who are under- or normal weight, particularly when they received no education or had higher educational level. As previously mentioned, obesity and physical inactivity are consistently linked to diabetes risk.[39] Younger people are thought to have high level of physical activity, yet the WHO in its Global report on diabetes indicated that high level of physical inactivity was observed among adolescents (84% of girls and 78% of boys).[39] Young adults also consume a lot of alcohol and tobacco, which are linked to increased diabetes prevalence.[50, 52] The reason why this was more common in participants with no education together with the ones having higher educational level particularly is not clear.

South Africa is one of the fastest growing economies around the world and ranked as an upper-middle-income country by the world bank.[4] In order to understand the reason for the increasing diabetes prevalence and the difference in the effect of risk factors compared to high-income countries, there are certain sociocultural and lifestyle factors that need to be considered. Though obesity is often linked to low SES in high-income country, in many African countries it is a sign of high SES and wealth. It is even seen as an indication for good health and beauty among females. Physical activity is also not preferable for females and seen as a masculine action in many cultures. It is also associated with performing sports not as a healthy lifestyle.[52].

Effect of urbanization and population aging is anticipated to cause the steady increase in diabetes prevalence, and is expected to be doubled by 2030.[60] Omran described the epidemiological transition as the change from high prevalence of infectious diseases associated with malnutrition, to high prevalence of chronic diseases associated with urban–industrial lifestyles.[61] Nutritional and physical activity transition due to demographic and economic factors has been described by Young et al., in five patterns. In the first pattern (hunter–gatherer population), diet consisted of high carbohydrates and fiber and low in fat, but high physical activity and minimal obesity. This gradually changes through the different patterns, until it reaches the fourth pattern where diet is high in total fat, cholesterol, sugar and other refined carbohydrates, and low in polyunsaturated fatty acids and fiber, associated with increase in sedentary life, leading to obesity and chronic diseases such as diabetes.[62] Nutrition transition is South Africa is due to urbanization and globalization, which lead to the consumption of energy-dense foods and sugary beverages.[59] Socioeconomic inequalities in diabetes were also identified regarding fruit and vegetable consumption. People with low SES reportedly eat less fruits and vegetables.[11, 50] Another factor that should be taken into account, is the high percentage of undiagnosed diabetes. As previously mentioned, 38% of people with diabetes in South Africa are undiagnosed. [11]

This study confirmed that there is morbidity expansion in diabetes in South Africa. Obesity and physical inactivity are the main contributing factors; however, understanding the predisposing circumstances for this is very important. Taking into consideration the aforementioned sociocultural and lifestyle factors, group-oriented prevention programs should take place with the right and convenient message that leads to better response and more compliance from each group. It is indicated in the literature, that moderate levels of physical activity could provide protection from certain chronic diseases including diabetes.[55] Providing suitable ways to increase physical activity and raising the awareness about the importance of eating a nutritional and healthy diet particularly in vulnerable groups is very essential. Furthermore, facilitating access, examination and treatment in health care centers to all groups of the society and especially people living in rural areas should be a priority.

## Limitations

There are some limitations that need to be considered while interpreting these data. The SADHS 2003 sample had an over-representation of urban areas and the African population group, along with an under-representation of whites and Indian females. Also, there seem to be data problems related to poor fieldwork, and inadequate training and supervision.[31] Over and under representation of some ethnicities might affect generalizability of such data within South Africa at that time point. Furthermore, the SADHS 2016 had some sampling and non-sampling errors which are common in most surveys.[32] Non-sampling errors such as failure to locate and interview the correct household, misunderstanding of the questions on the part of either the interviewer or the respondent, and data entry errors can only be minimized but are inevitable in any survey. Sampling errors, however, can be evaluated statistically. Another consideration is participants age range, which was between 15 and 95 years. This age range is different from some studies that were done in the same field and were used for comparison. Then again, dividing the participants in three different age groups, makes certain age-group targeted comparison easier. Additionally, certain variables were not available in both datasets, such as “wealth index” and “weight perception,” which limited the comparison between the two time points. Occupation was also not included in the analysis, because it was not clearly defined in both datasets. Furthermore, it was not specified whether participants had diabetes type 1 or type 2. Given that people with type 1 diabetes are estimated to be 7 to 12% of total diabetes population,[32] participants with diabetes in this study were considered to be having type 2 diabetes. Another possible limitation is that data were based on self-reported diabetes not based on examination, which could be subjected to recall bias. Nonetheless it is a widely used method in surveys and proved its reliability.

## Conclusion

There was an increase in diabetes prevalence in South Africa between 2003 and 2016. Socioeconomic inequalities were recognized at both time points. Main risk factors for developing diabetes were age, gender (women were more at risk), and living in urban areas. Educational level effect was not consistent in 2003 and for women, however, men with high educational level were more at risk of developing diabetes in 2016. Vulnerable groups in 2016 were participants 40 years and older, where risk of developing diabetes increased with increasing age and SES (high wealth-index). Participants younger than 40 years who were obese or overweight were more at risk of developing diabetes compared to their counterparts from the same age.

## Data Availability

The 2016 dataset was available without restriction. Researchers can gain access to datasets used for the analyses of this study through registration as DHS data user in this webpage: (The DHS Program - login_main.) www.dhsprogram.com/data/dataset_admin/login_main.cfm. The 2003 DHS was not published. DHS team were contacted and Dr Tshilidzi Muthivhi (Health Research Directorate. Department of Health, South Africa) sent an email with an attachment containing the 2003 dataset.

## List of abbreviations

IDF: International Diabetes Federation
NCDs: non-communicable diseases
DHS: Demographic and Health Surveys
SES: socioeconomic status
LMIC: low- and middle-income countries
SADHS: South Africa DHS
Stats SA: Statistics South Africa
EAs: enumeration areas
PSUs: primary sampling units
DUs: dwelling units
ORs: Odds Ratios
CIs: Confidence Intervals
CART: Classification and Regression Tree
RODAM: Research on Obesity and Diabetes among African Migrants
